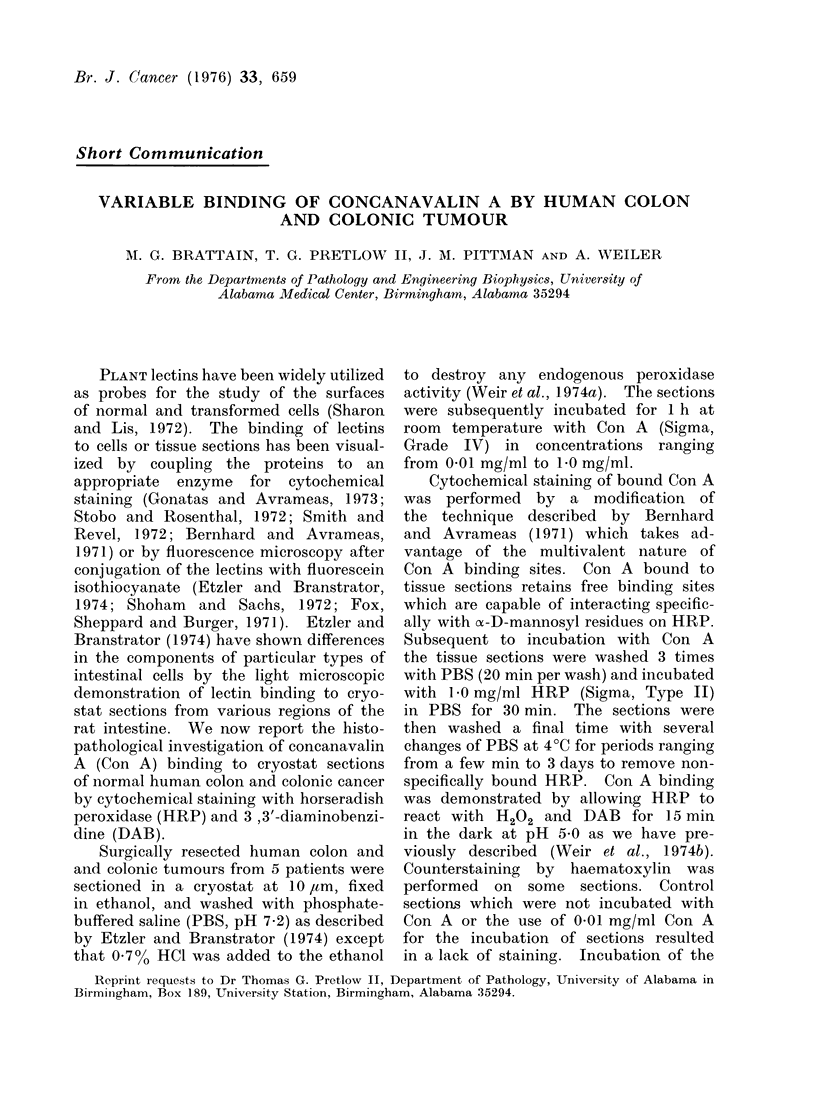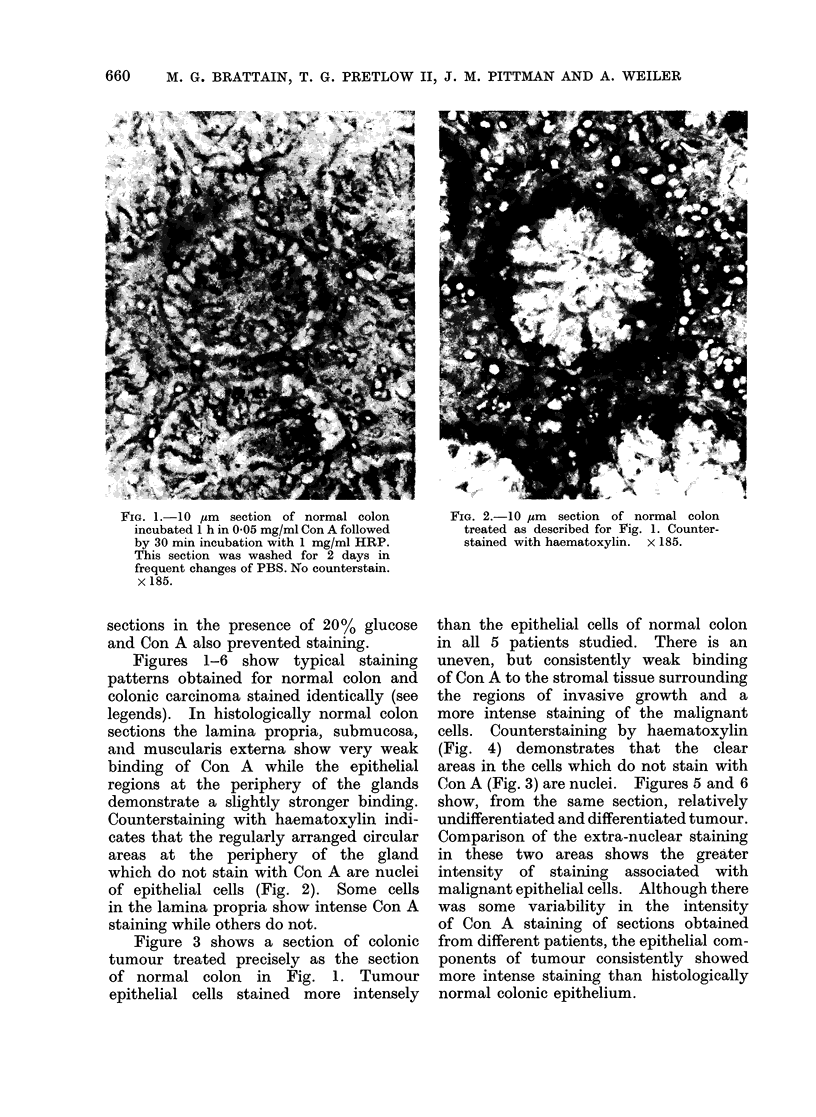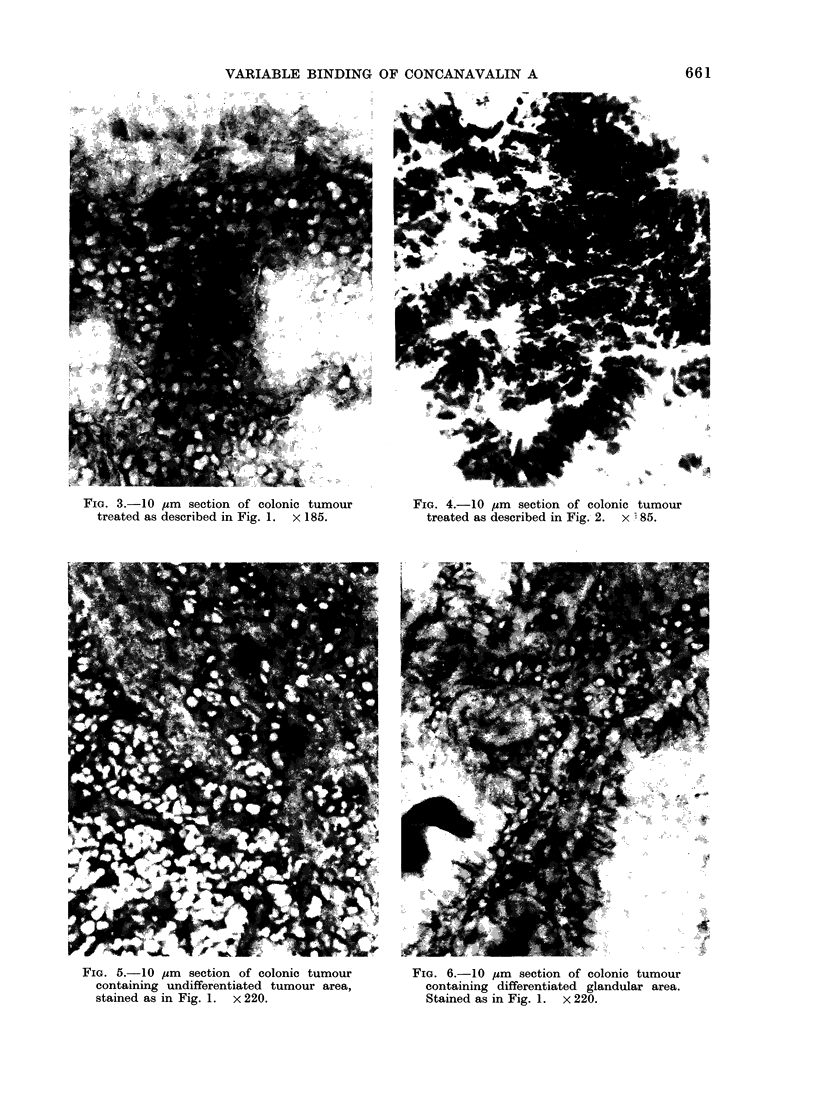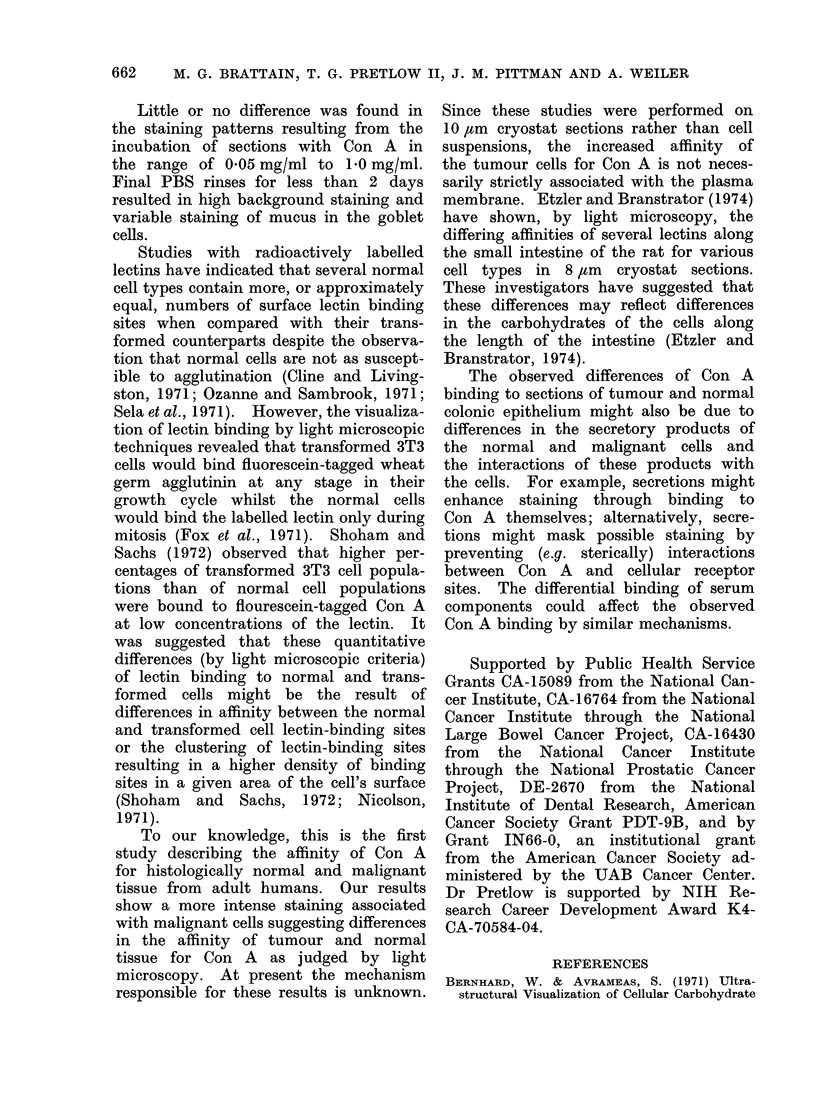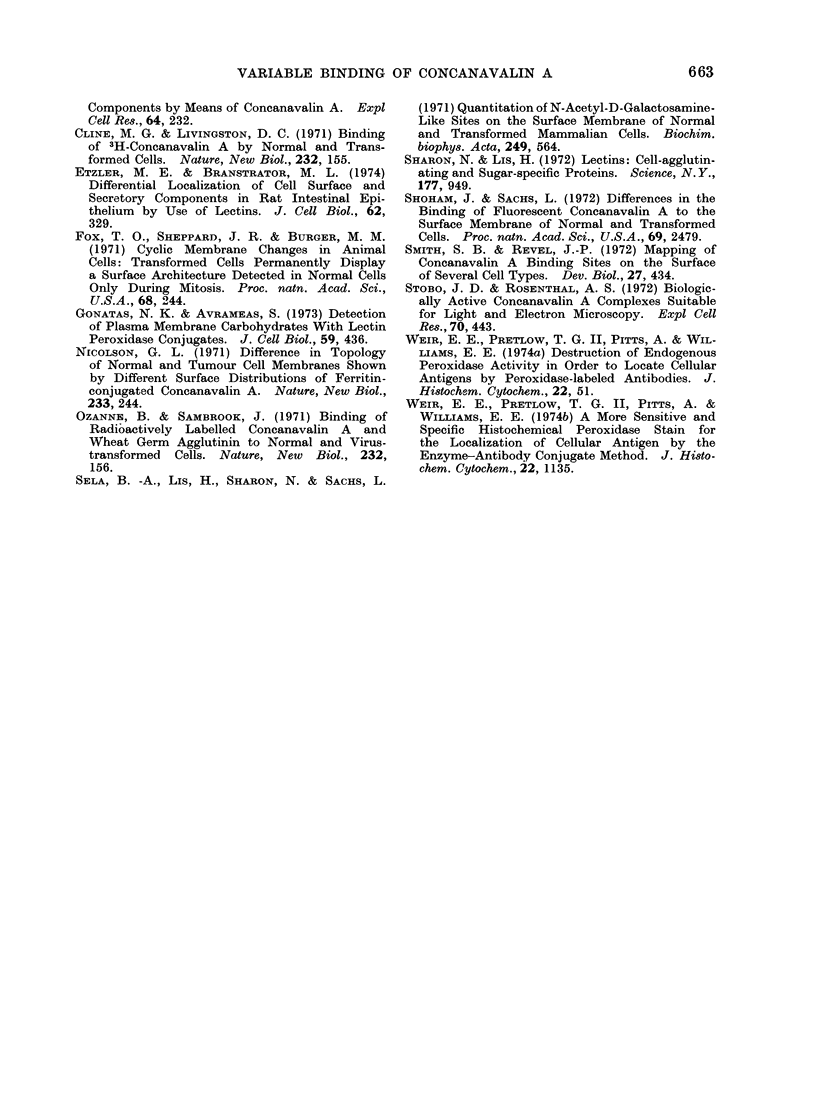# Variable binding of concanavalin A by human colon and colonic tumour.

**DOI:** 10.1038/bjc.1976.104

**Published:** 1976-06

**Authors:** M. G. Brattain, T. G. Pretlow, A. Weiler

## Abstract

**Images:**


Br. J. Cancer (1976) 33, 659

### Short Communication

### VARIABLE BINDING OF CONCANAVALIN A BY HUMAN COLON AND COLONIC TUMOUR

M. G. BRATTAIN, T. G. PRETLOW II, J. M. PITTMAN AND A. WEILER

*From the Departments of Pathology and Engineering Biophysics, University of Alabama Medical Center, Birmingham, Alabama 35294*

PLANT lectins have been widely utilized as probes for the study of the surfaces of normal and transformed cells (Sharon and Lis, 1972). The binding of lectins to cells or tissue sections has been visualized by coupling the proteins to an appropriate enzyme for cytochemical staining (Gonatas and Avrameas, 1973; Stobo and Rosenthal, 1972; Smith and Revel, 1972; Bernhard and Avrameas, 1971) or by fluorescence microscopy after conjugation of the lectins with fluorescein isothiocyanate (Etzler and Branstrator, 1974; Shoham and Sachs, 1972; Fox, Sheppard and Burger, 1971). Etzler and Branstrator (1974) have shown differences in the components of particular types of intestinal cells by the light microscopic demonstration of lectin binding to cryostat sections from various regions of the rat intestine. We now report the histopathological investigation of concanavalin A (Con A) binding to cryostat sections of normal human colon and colonic cancer by cytochemical staining with horseradish peroxidase (HRP) and 3 ,3′-diaminobenzidine (DAB).

Surgically resected human colon and and colonic tumours from 5 patients were sectioned in a cryostat at 10 $\mu$m, fixed in ethanol, and washed with phosphate-buffered saline (PBS, pH 7·2) as described by Etzler and Branstrator (1974) except that 0·7% HCl was added to the ethanol to destroy any endogenous peroxidase activity (Weir *et al.*, 1974*a*). The sections were subsequently incubated for 1 h at room temperature with Con A (Sigma, Grade IV) in concentrations ranging from 0·01 mg/ml to 1·0 mg/ml.

Cytochemical staining of bound Con A was performed by a modification of the technique described by Bernhard and Avrameas (1971) which takes advantage of the multivalent nature of Con A binding sites. Con A bound to tissue sections retains free binding sites which are capable of interacting specifically with α-D-mannosyl residues on HRP. Subsequent to incubation with Con A the tissue sections were washed 3 times with PBS (20 min per wash) and incubated with 1·0 mg/ml HRP (Sigma, Type II) in PBS for 30 min. The sections were then washed a final time with several changes of PBS at 4°C for periods ranging from a few min to 3 days to remove non-specifically bound HRP. Con A binding was demonstrated by allowing HRP to react with $H_2O_2$ and DAB for 15 min in the dark at pH 5·0 as we have previously described (Weir *et al.*, 1974*b*). Counterstaining by haematoxylin was performed on some sections. Control sections which were not incubated with Con A or the use of 0·01 mg/ml Con A for the incubation of sections resulted in a lack of staining. Incubation of the

Reprint requests to Dr Thomas G. Pretlow II, Department of Pathology, University of Alabama in Birmingham, Box 189, University Station, Birmingham, Alabama 35294.

FIG. 1.—10 $\mu$m section of normal colon incubated 1 h in 0·05 mg/ml Con A followed by 30 min incubation with 1 mg/ml HRP. This section was washed for 2 days in frequent changes of PBS. No counterstain. ×185.

FIG. 2.—10 $\mu$m section of normal colon treated as described for Fig. 1. Counterstained with haematoxylin. ×185.

sections in the presence of 20% glucose and Con A also prevented staining.

Figures 1–6 show typical staining patterns obtained for normal colon and colonic carcinoma stained identically (see legends). In histologically normal colon sections the lamina propria, submucosa, and muscularis externa show very weak binding of Con A while the epithelial regions at the periphery of the glands demonstrate a slightly stronger binding. Counterstaining with haematoxylin indicates that the regularly arranged circular areas at the periphery of the gland which do not stain with Con A are nuclei of epithelial cells (Fig. 2). Some cells in the lamina propria show intense Con A staining while others do not.

Figure 3 shows a section of colonic tumour treated precisely as the section of normal colon in Fig. 1. Tumour epithelial cells stained more intensely than the epithelial cells of normal colon in all 5 patients studied. There is an uneven, but consistently weak binding of Con A to the stromal tissue surrounding the regions of invasive growth and a more intense staining of the malignant cells. Counterstaining by haematoxylin (Fig. 4) demonstrates that the clear areas in the cells which do not stain with Con A (Fig. 3) are nuclei. Figures 5 and 6 show, from the same section, relatively undifferentiated and differentiated tumour. Comparison of the extra-nuclear staining in these two areas shows the greater intensity of staining associated with malignant epithelial cells. Although there was some variability in the intensity of Con A staining of sections obtained from different patients, the epithelial components of tumour consistently showed more intense staining than histologically normal colonic epithelium.

FIG. 3.—10 μm section of colonic tumour treated as described in Fig. 1. ×185.

FIG. 4.—10 μm section of colonic tumour treated as described in Fig. 2. ×185.

FIG. 5.—10 μm section of colonic tumour containing undifferentiated tumour area, stained as in Fig. 1. ×220.

FIG. 6.—10 μm section of colonic tumour containing differentiated glandular area. Stained as in Fig. 1. ×220.

Little or no difference was found in the staining patterns resulting from the incubation of sections with Con A in the range of 0·05 mg/ml to 1·0 mg/ml. Final PBS rinses for less than 2 days resulted in high background staining and variable staining of mucus in the goblet cells.

Studies with radioactively labelled lectins have indicated that several normal cell types contain more, or approximately equal, numbers of surface lectin binding sites when compared with their transformed counterparts despite the observation that normal cells are not as susceptible to agglutination (Cline and Livingston, 1971; Ozanne and Sambrook, 1971; Sela *et al.*, 1971). However, the visualization of lectin binding by light microscopic techniques revealed that transformed 3T3 cells would bind fluorescein-tagged wheat germ agglutinin at any stage in their growth cycle whilst the normal cells would bind the labelled lectin only during mitosis (Fox *et al.*, 1971). Shoham and Sachs (1972) observed that higher percentages of transformed 3T3 cell populations than of normal cell populations were bound to flourescein-tagged Con A at low concentrations of the lectin. It was suggested that these quantitative differences (by light microscopic criteria) of lectin binding to normal and transformed cells might be the result of differences in affinity between the normal and transformed cell lectin-binding sites or the clustering of lectin-binding sites resulting in a higher density of binding sites in a given area of the cell's surface (Shoham and Sachs, 1972; Nicolson, 1971).

To our knowledge, this is the first study describing the affinity of Con A for histologically normal and malignant tissue from adult humans. Our results show a more intense staining associated with malignant cells suggesting differences in the affinity of tumour and normal tissue for Con A as judged by light microscopy. At present the mechanism responsible for these results is unknown. Since these studies were performed on 10 $\mu$m cryostat sections rather than cell suspensions, the increased affinity of the tumour cells for Con A is not necessarily strictly associated with the plasma membrane. Etzler and Branstrator (1974) have shown, by light microscopy, the differing affinities of several lectins along the small intestine of the rat for various cell types in 8 $\mu$m cryostat sections. These investigators have suggested that these differences may reflect differences in the carbohydrates of the cells along the length of the intestine (Etzler and Branstrator, 1974).

The observed differences of Con A binding to sections of tumour and normal colonic epithelium might also be due to differences in the secretory products of the normal and malignant cells and the interactions of these products with the cells. For example, secretions might enhance staining through binding to Con A themselves; alternatively, secretions might mask possible staining by preventing (*e.g.* sterically) interactions between Con A and cellular receptor sites. The differential binding of serum components could affect the observed Con A binding by similar mechanisms.

Supported by Public Health Service Grants CA-15089 from the National Cancer Institute, CA-16764 from the National Cancer Institute through the National Large Bowel Cancer Project, CA-16430 from the National Cancer Institute through the National Prostatic Cancer Project, DE-2670 from the National Institute of Dental Research, American Cancer Society Grant PDT-9B, and by Grant IN66-0, an institutional grant from the American Cancer Society administered by the UAB Cancer Center. Dr Pretlow is supported by NIH Research Career Development Award K4-CA-70584-04.

### REFERENCES

Bernhard, W. & Avrameas, S. (1971) Ultrastructural Visualization of Cellular Carbohydrate

Components by Means of Concanavalin A. *Expl Cell Res.*, **64**, 232.
Cline, M. G. & Livingston, D. C. (1971) Binding of $^3$H-Concanavalin A by Normal and Transformed Cells. *Nature, New Biol.*, **232**, 155.
Etzler, M. E. & Branstrator, M. L. (1974) Differential Localization of Cell Surface and Secretory Components in Rat Intestinal Epithelium by Use of Lectins. *J. Cell Biol.*, **62**, 329.
Fox, T. O., Sheppard, J. R. & Burger, M. M. (1971) Cyclic Membrane Changes in Animal Cells: Transformed Cells Permanently Display a Surface Architecture Detected in Normal Cells Only During Mitosis. *Proc. natn. Acad. Sci., U.S.A.*, **68**, 244.
Gonatas, N. K. & Avrameas, S. (1973) Detection of Plasma Membrane Carbohydrates With Lectin Peroxidase Conjugates. *J. Cell Biol.*, **59**, 436.
Nicolson, G. L. (1971) Difference in Topology of Normal and Tumour Cell Membranes Shown by Different Surface Distributions of Ferritin-conjugated Concanavalin A. *Nature, New Biol.*, **233**, 244.
Ozanne, B. & Sambrook, J. (1971) Binding of Radioactively Labelled Concanavalin A and Wheat Germ Agglutinin to Normal and Virus-transformed Cells. *Nature, New Biol.*, **232**, 156.
Sela, B. -A., Lis, H., Sharon, N. & Sachs, L. (1971) Quantitation of N-Acetyl-D-Galactosamine-Like Sites on the Surface Membrane of Normal and Transformed Mammalian Cells. *Biochim. biophys. Acta*, **249**, 564.
Sharon, N. & Lis, H. (1972) Lectins: Cell-agglutinating and Sugar-specific Proteins. *Science, N.Y.*, **177**, 949.
Shoham, J. & Sachs, L. (1972) Differences in the Binding of Fluorescent Concanavalin A to the Surface Membrane of Normal and Transformed Cells. *Proc. natn. Acad. Sci., U.S.A.*, **69**, 2479.
Smith, S. B. & Revel, J.-P. (1972) Mapping of Concanavalin A Binding Sites on the Surface of Several Cell Types. *Dev. Biol.*, **27**, 434.
Stobo, J. D. & Rosenthal, A. S. (1972) Biologically Active Concanavalin A Complexes Suitable for Light and Electron Microscopy. *Expl Cell Res.*, **70**, 443.
Weir, E. E., Pretlow, T. G. II, Pitts, A. & Williams, E. E. (1974*a*) Destruction of Endogenous Peroxidase Activity in Order to Locate Cellular Antigens by Peroxidase-labeled Antibodies. *J. Histochem. Cytochem.*, **22**, 51.
Weir, E. E., Pretlow, T. G. II, Pitts, A. & Williams, E. E. (1974*b*) A More Sensitive and Specific Histochemical Peroxidase Stain for the Localization of Cellular Antigen by the Enzyme–Antibody Conjugate Method. *J. Histochem. Cytochem.*, **22**, 1135.